# Adherence to treatment in children with growth hormone deficiency, small for gestational age and Turner syndrome in Mexico: results of the Easypod™ connect observational study (ECOS)

**DOI:** 10.1007/s40618-020-01218-4

**Published:** 2020-04-01

**Authors:** A. Blanco-López, C. Antillón-Ferreira, E. Saavedra-Castillo, M. Barrientos-Pérez, H. Rivero-Escalante, O. Flores-Caloca, R. Calzada-León, C. C. Rosas-Guerra, E. Koledova, E. Chiquete, A. Ayala-Estrada

**Affiliations:** 1 Hospital Ángeles Interlomas and Hospital Español, Mexico City, Mexico; 2 Hospital Español, Mexico City, Mexico; 3 Hospital de la Oca, Monterrey, Nuevo León Mexico; 4 Hospital Ángeles, Puebla, Puebla, Mexico; 5 Private Practice, Cancún, Quintana Roo Mexico; 6 Hospital Zambrano Hellion, Monterrey, Nuevo León Mexico; 7 Servicio de Endocrinología, Instituto Nacional de Pediatría, Mexico City, Mexico; 8 Merck Biopharma Distribution S.A. de C.V, Naucalpan de Juárez, Mexico; 9 Merck Healthcare KGaA, Darmstadt, Germany; 10 Departamento de Neurología y Psiquiatría, Instituto Nacional de Ciencias Médicas y Nutrición Salvador Zubirán, Delegacion Tlalpan, C.P. 14080, Ciudad de Mexico, México; 11 Servicio de Pediatría, Hospital ISSEMyM, Toluca, Estado de México México

**Keywords:** Adherence, Auto-injector, Device, Growth hormone, Somatotropin

## Abstract

**Background:**

Assessing adherence to growth hormone (GH) is challenging. The Easypod™ connect device delivers pre-set doses of recombinant human GH (r-hGH) and stores a digital record of adherence that can be shared with healthcare provider. We assessed adherence to r-hGH delivered with Easypod™ according to the approved pediatric indications for r-hGH: growth hormone deficiency (GHD), born small for gestational age (SGA) who failed to show catch-up growth and Turner syndrome (TS).

**Methods:**

ECOS (NCT01555528) was a multicenter (24 countries), 5-year, longitudinal, observational study, which aimed to evaluate country-specific adherence to r-hGH therapy prescribed via the Easypod™ electronic injection device. The primary endpoint was yearly adherence. Secondary endpoints were height velocity, height velocity standard deviation scores (SDS), height, height SDS and IGF-1 concentrations. Clinical and auxological data were obtained from medical records and adherence from Easypod™ logs.

**Results:**

This study included 147 Easypod™-naïve Mexican children assessed during 3 years (mean age: 9.96 ± 3.41 years, 56.8% boys, mean height SDS at baseline: − 2.17 ± 0.97): 118 with GHD, 24 SGA and 5 with TS. A total of 105 (71.4%) patients were GH naïve. Overall median adherence was > 90% over the first year of treatment and > 80% at 3 years. Adherence was not different by r-hGH indication or between GH-naïve or experienced patients. At 1-year follow-up, mean change in height SDS was 0.57 ± 0.34, whereas mean height velocity SDS was 2.85 ± 2.51. In all, 84.7% patients had normal IGF-1 concentrations at 1-year follow-up. Adherence was associated with change in height SDS (*r* = 0.239, *p* = 0.005) and height velocity SDS (*r* = 0.194, *p* = 0.027).

**Conclusion:**

Adherence rates with the Easypod™ device are high and maintained over time in GHD, SGA and TS Easypod™-naïve Mexican patients. High adherence is associated with better outcomes. Easypod™ assists physicians in monitoring adherence to r-hGH.

## Introduction

The human recombinant growth hormone (h-rGH) is indicated to treat clinical conditions that imply short stature, such as GH deficiency (GHD), children born small for gestational age (SGA) who failed to show catch-up growth and Turner syndrome (TS), among other medical conditions [1–6]. Adherence to h-rGH is one of the most important factors that determine achievement of clinical targets [7−10]. Unfortunately, adherence to GH therapy has been reported suboptimal in the majority of patients [11−13]. But an additional barrier is actual recognition of adherence by healthcare providers due to patients and/or family under reporting [6]. Hence, detection of poor adherence can be challenging in real-life settings.

The Easypod^TM^ auto-injector connected digital device (approved in Mexico in June 2016) is designed to make daily administration of recombinant human growth hormone (r-hGH) comfortable and easier to patients. Easypod^TM^ device delivers pre-set doses of r-hGH (Saizen®) and stores a digital record of adherence to therapy that can be electronically shared with healthcare providers for evaluation [5, 6, 13−21]. Furthermore, Easypod^TM^ is integrated into an e-Health ecosystem for management of growth disorders treated with Saizen (r-hGH) available to healthcare professionals through a personal-data secure web solution.

The aim of the present report was to assess adherence to r-hGH therapy delivered via the Easypod^TM^ device according to the approved pediatric indications for Saizen® in Mexico: GHD, SGA and TS. A secondary objective of the present analysis is to evaluate the potential association of adherence with growth outcomes.

## Methods

The Easypod^TM^ connect observational study (ECOS, NCT01555528) was multicenter (24 countries), 5-year (November 2010–February 2016), phase IV, prospective, longitudinal and observational study, to assess country-specific adherence to therapy among children receiving r-hGH via the Easypod^TM^ electromechanical auto-injector device. Herein, we present the subanalysis for the Mexican population included in ECOS. The study was performed in accordance with the principles of the Declaration of Helsinki, Good Clinical Practice (ICH-GCP E6) guidelines and applicable local regulatory requirements. A central Institutional Review Board and Ethics Committee in Mexico approved this part of the study. All patients agreed for participating in the study and their parents or legal proxies provided signed informed consent.

Methods of ECOS have already been reported elsewhere [6]. To be eligible, patients should be EasypodTM naïve but could be experienced with r-hGH delivered by other methods. Briefly, Easypod^TM^-naïve children included in the study were aged 2–18 years or >18 years without fusion of growth plates and were receiving r-hGH via the Easypod^TM^ electromechanical device (Saizen®, Merck KGaA, Darmstadt, Germany). Eligible patients from each participating country were enrolled and attended one baseline visit followed by 1–4 visits each year, at healthcare provider discretion and according to local clinical practice. Also, all diagnoses and treatment decisions were at the discretion of the researcher physician.

The primary endpoint was the recorded adherence as assessed at yearly intervals. Secondary endpoints were height velocity, height velocity standard deviation scores (SDS), height, height SDS, as well as IGF-1 concentrations after each year of treatment. Demographic, auxological and diagnostic data were obtained from medical records, with adherence data obtained directly from the patients’ Easypod^TM^ electronic records.

Relative frequencies are expressed as percentages. Parametric continuous variables are expressed as means and standard deviations (SD). Non-parametric continuous variables will be expressed as medians with minimum and maximum or interquartile range. Pearson Chi-square or Fisher exact tests are used to assess proportions in categorical variables. To compare quantitative variables between two groups, Student *t* test and Mann–Whitney *U* test were performed in distributions of parametric and non-parametric variables, respectively. Adherence was determined as the percentage adherence over time (number of days with injections received divided by the number of days with injections planned). Correlations between adherence and growth outcomes were calculated using Spearman’s product–moment correlation. Alpha errors (*p* values) reported are two sided and considered significant when *p* < 0.05.

## Results

ECOS included 147 Mexican patients (mean age: 9.96 ± 3.41 years, 56.8% boys, mean height SDS at baseline: − 2.17 ± 0.97): 118 with GHD, 24 SGA patients who failed to show catch-up growth and 5 with TS (Table 1). A total of 105 (71.4%) patients were GH naïve. Mean age was not significantly different among groups, however, with a trend for patients with TS being younger (Table 2).

**Table 1:** Baseline characteristics of the cohort (*n* = 147)

	GHD (*n* = 118)	SGA (*n* = 24)	TS (*n* = 5)	Overall (*n* = 147)
Age (years)				
Mean (SD)	9.95 (3.52)	10.13 (2.40)	8.60 (4.88)	9.96 (3.41)
Median	10.5	10	10	10
Min; max	1; 18	5; 15	3; 14	1; 18
Sex, *n* (%)				
Female	50 (42.4)	9 (37.5)	5 (100)	64 (43.2)
Male	68 (57.6)	15 (62.5)	0	84 (56.8)
Ethnicity, *n* (%)				
Caucasian	5 (4.2)	1 (4.2)	0	6 (4.1)
Other	113 (95.8)	23 (95.8)	5 (100)	142 (95.9)

*GHD* growth hormone deficiency, *SGA* small for gestational age patient who failed to show catch-up growth, *TS* Turner syndrome

**Table 2:** Growth outcomes at 1-year follow-up (*n* = 147)

	GHD (*n* = 118)	SGA (*n* = 24)	TS (*n* = 5)	Overall (*n* = 147)
Height SDS at baseline				
Mean (SD)	− 2.17 (0.98)	− 1.93 (0.76)	− 3.36 (1.06)	− 2.17 (0.97)
Median (IQR)	− 2.06 (−2.61 to − 1.72)	− 1.89 (−2.43 to −1.55)	− 3.10 (−3.79 to −2.58)	− 2.08 (−2.61 to −1.72)
Change in height SDS				
Mean (SD)	0.58 (0.35)	0.51 (0.30)	0.63 (0.42)	0.57 (0.34)
Median (IQR)	0.56 (0.36–0.76)	0.44 (0.32–0.76)	0.80 (0.40–0.88)	0.54 (0.36–0.77)
Height velocity (cm per year) SDS				
Mean (SD)	2.97 (2.62)	2.64 (1.97)	0.64 (1.65)	2.85 (2.51)
Median (IQR)	3.04 (1.58–4.17)	2.65 (1.54–3.71)	1.05 (0.60–1.88)	2.91 (1.49–4.07)
1-year IGF-1 standard score (%)				
Abnormal low (< 84 µ/L)	9.6	16.7	0	10.2
Normal (84–100 µ/L)	86.5	66.7	100	84.7
Abnormal high (> 100 µ/L)	3.8	16.7	0	5.1

*GHD* growth hormone deficiency, *SDS* standard deviation scores, *SGA* small for gestational age patient who failed to show catch-up growth, *TS* Turner syndrome

**Fig. 1:**
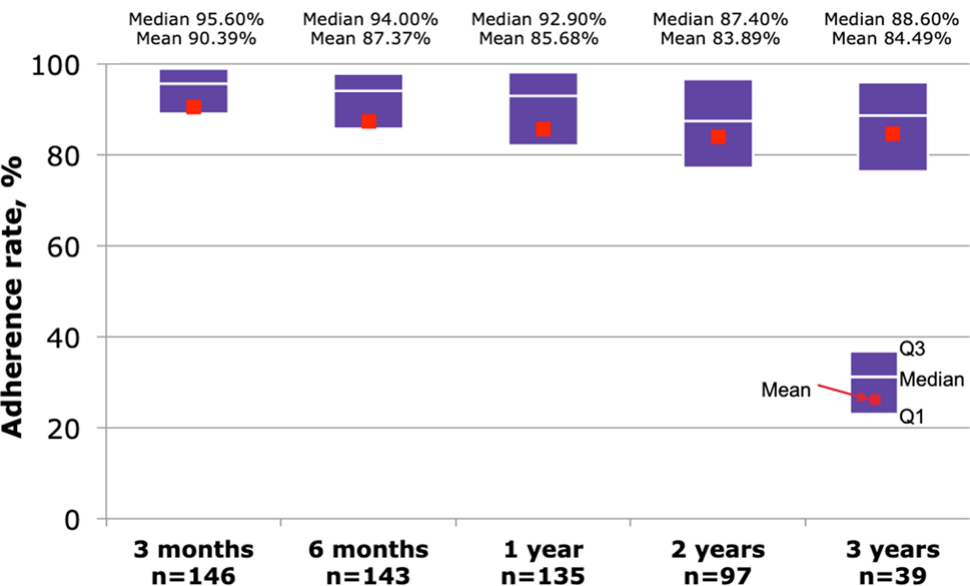
Adherence to r-hGH therapy delivered via the Easypod™ device from baseline through year 3 follow-up

**Fig. 2:**
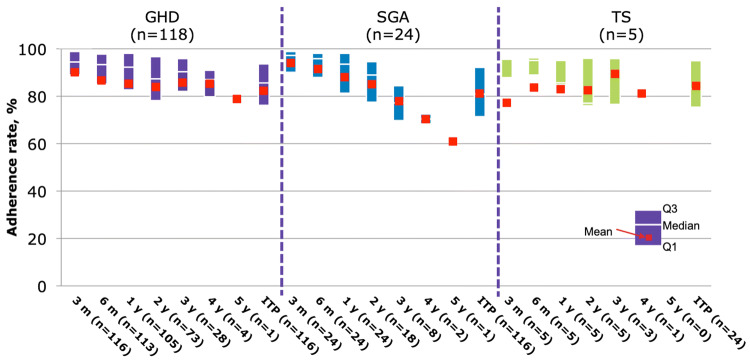
Adherence to r-hGH therapy delivered via the Easypod™ device, according to diagnosis. *GHD* Growth hormone deficiency, *SGA* small for gestational age patient who failed to show catch-up growth, *TS* Turner syndrome

Overall median adherence was >90% over the first year of treatment and >80% over 3 years (Fig. 1); however, as this was an observational study in Mexico and most patients were treated privately significant study dropouts where observed after the first year of therapy on patients enrolled the study. Adherence was not different by r-hGH indication of between GH-naïve or experienced patients (Fig. 2). At 1-year follow-up, mean change in height SDS was 0.57 ± 0.34, whereas mean height velocity SDS was 2.85 ± 2.51.

At 1-year follow-up, 84.7% patients had normal IGF-1 concentrations (84–100 µ/L), 10.2% had abnormal low (<84 µ/L) and 5.1% abnormal high IGF-1 concentrations (>100 µ/L). Overall, statistically significant correlations were observed with therapy adherence and change in height SDS (*p* = 0.005), as well as with height velocity SDS (*p* = 0.027) (Table 3). Nonetheless, correlation of treatment adherence with change in height SDS was lower and not significant in the GH-naïve subgroup (Spearman’s product–moment correlation = 0.147, *p* = 0.134).

**Table 3:** Spearman’s product–moment correlation of therapy adherence with growth outcomes at 1-year follow-up

	GHD (*n* = 118)	SGA (*n* = 24)	TS (*n* = 5)	Overall (*n* = 147)
Change in height SDS				
Spearman's rho	0.215	0.376	− 0.500	0.239
*p* value	0.028	0.070	0.391	0.005
Height velocity SDS				
Spearman's rho	0.187	0.184	0.200	0.194
*p* value	0.061	0.390	0.800	0.027

*GHD* growth hormone deficiency, *SDS* standard deviation scores, *SGA* small for gestational age patient who failed to show catch-up growth, *TS* Turner syndrome

## Discussion

The present analysis shows that adherence to r-hGH delivered by the Easypod™ electromechanical auto-injection device in Mexican children with different causes of short stature remains high after 3 years of treatment. Furthermore, the magnitude of adherence was directly associated with anthropometric and auxological milestones during r-hGH therapy. Although we cannot discard the Hawthorne effect (i.e., the reactivity in which individuals modify an aspect of their behavior in response to their awareness of being observed), our data and those of the global ECOS report [6] suggest that children under r-hGH therapy delivered by the Easypod™ device are prone to present a high rate of adherence in the mid- and long terms.

In the present study, the observed adherence rate was above 80% up to 3 years of clinical follow-up, which contrasts with the expected adherence rate of 55–65% observed in other studies using different r-hGH delivery technologies [22, 23] and confirms the findings of other reports with the same methodology [5, 6, 13, 14, 24]. Additionally, this study suggests that adherence to therapy is predictor of clinical response, a factor that has been described in other scenarios where electromechanical auto-injectors are used to deliver drugs and to assess compliance to therapy [25−30]. As can be inferred, poor adherence is usually associated with failure to reaching clinical outcomes [6, 13]. The global ECOS study found a positive correlation between adherence and attainment of growth milestones [6]. Contrary to previous reports [5, 13], here, we found a significant correlation between adherence and growth outcomes at 1-year after initiating r-hGH therapy with Easypod™. This difference among ECOS subpopulations may be due to sample size differences as well as the proportions of r-hGH therapy indications, which may lead to potential differences in the magnitude of clinical response.

A limitation of this study, however, is the low sample of patients that remained after 3 years of follow-up, which make difficult assumptions regarding very long-term adherence, associated factors and attainment of clinical objectives of the r-hGH therapy in the long run. Moreover, determinants of adherence such as socioeconomical status, parental education status, achievement of therapeutic goals and patient’s perspective about therapy were not taken into account for the present analysis. Side effects associated with therapy were not analyzed since in this cohort as global ECOS data did not show any new safety findings [6] and the main objective of this study was to assess adherence to an electronic auto-injector device in real-world settings in Mexican patients. Although efficacy was not an objective of this study, we observed attainment of clinical milestones of r-hGH therapy as can be expected by clinical indication. Nevertheless, this ECOS subanalysis on Mexican patients demonstrates that adherence to therapy is high with Easypod™ device and that adherence relates with clinical response.

## Conclusion

In conclusion, ECOS has produced robust, real-world adherence data in patients receiving Saizen^®^ via Easypod™ and provided useful insights into growth response to Saizen^®^ treatment. Adherence rates with the Easypod^TM^ device are high and maintained over time in GHD and SGA Mexican patients. The positive correlations between adherence and growth outcomes suggest an influence of adherence on treatment outcomes. Nonetheless, this may also be influenced by the fact that in Mexico, most patients were treated privately, have regular consultations and received a slightly higher mean dose of r-hGH, as compared with other countries of the ECOS study.
